# Male Reproduction: Regulation, Differentiation and Epigenetics

**DOI:** 10.3390/genes13061001

**Published:** 2022-06-02

**Authors:** Massimo Venditti, Sergio Minucci

**Affiliations:** Dipartimento di Medicina Sperimentale, Sez. Fisiologia Umana e Funzioni Biologiche Integrate, Università degli Studi della Campania ‘Luigi Vanvitelli’, Via Santa Maria di Costantinopoli, 16-80138 Napoli, Italy

The production of good-quality spermatozoa (SPZ) is one of the most intricate and far from being completely understood developmental processes during postnatal life. Indeed, it is broadly recognized that male fertility depends on a certain amount of SPZ with proper concentration, morphology, motility, and DNA integrity [1]. The intrinsic complexity of the process lies in the fact that it is controlled by cell type- and stage-specific genes, and by several central and local modulators. For this, spermatogenesis is extremely delicate, as all the phases leading to the differentiation of mature SPZ (spermatogenesis, spermiogenesis and their hormonal control) can be disturbed by either endogenous (genetic issues, pathologies, and hypogonadism) and exogenous (environmental pollution and lifestyle) factors. Nowadays, a progressive reduction in gamete quality is occurring, and infertility is one of the most important concerns in the industrialized world, mostly due to the impact that pollutants have on the different phases of spermatogenesis [2,3]. Therefore, there is a necessity to expand the knowledge on the mechanism(s) of male gamete differentiation, with the aim to increase the plethora of possible markers and therapeutic targets in the clinical setting. This Special Issue collects a total of seven articles, four review articles and three research articles, and expands the current knowledge on male reproduction and fertility in both physiological and pathological conditions.

Mele et al. [4] characterized the kisspeptin system in rat SPZ and epididymis caput and cauda and analyzed the possible presence of Kiss1 in the epididymal fluid. Indeed, though the activity of the kisspeptin system has been studied at the central and local levels, the need of peripheral kisspeptin to produce gametes is not fully understood. They reported, for the first time in rodents, Kiss1R trafficking in SPZ during the epididymis transit, suggesting a possible role for the system in gamete maturation and storage within the epididymis.

Venditti et al. [5] studied the effects of the alterations induced by cadmium on germ cell cytoarchitecture, and the putative counteractive action posed by melatonin. In particular, they confirmed that cadmium increased oxidative stress and apoptosis of germ and somatic cells and reduced testosterone bioavailability. In addition, they also found a reduced expression and altered localization of two cytoskeleton-associated proteins, DAAM1 and PREP, which are involved in the germ cells’ differentiation into SPZ. Finally, the co-treatment with melatonin attenuated all the alterations induced by cadmium, encouraging further studies to prove its effectiveness in human health.

Wu et al. [6] provided an interesting paper on the evolution of reproductive life history in mammals. They reconstructed the evolutionary history of several continuous traits (such as lifespan and bodyweight), and five reproduction-related traits via a regression analysis, using the fitted model to predict ancestral states and to obtain the genes that may be related to the traits.

One of the of major risk factors with impact on testis anatomy and histology, impairing the reproductive function, is cryptorchidism (undescended testis) and the paper by Ciongradi et al. [7] analyzed its correlation with fertility. Indeed, cryptorchidism is one of the most common diagnoses in the pediatric age. In this review, the authors focused their attention on the efforts that should be made to preserve germ cell development in young patients affected by cryptorchidism, such as the hormonal therapy and the development of studies focused on spermatogonia stem cells transplantation.

The importance of fertility preservation is fundamental also for pediatric and adolescent oncologic patients, as reviewed by Bîcă et al. [8]. Indeed, one of the most common side-effects of cancer treatments (chemo- and radiotherapies) is their impacts on fertility, thus the importance of techniques aimed at fertility preservation (as sperm and testicular tissue cryopreservation, TESE, gonadal shielding, and stem cells transplantation) is of primary importance. 

As stated above, for good fertility, SPZ with a proper morphology are essential. In their review, Manfrevola et al. [9] focused their attention on the role of the LINC (linker of nucleoskeleton and cytoskeleton) complex, a nuclear envelope-bridge structure involved in the connection of the nucleoskeleton to the cytoskeleton, during spermiogenesis. They extensively discussed the role of LINC complex components in the SPZ head formation and head to tail connection and provided an overview on the association between LINC complex alterations and male infertility. 

Finally, Slater and colleagues [10] placed their attention on the fertility of male honeybees, a specie that has fundamental ecological and economical importance, but that, at the same time, is worryingly threatened by anthropic activities. They discussed the up-to-date knowledge of how genetic variation within and between populations contributes to variation in SPZ production and maintenance, as well as insemination success among male bees. 

In conclusion, this research topic provides an updated contribution to the subject of male reproduction. The papers indicated the need to clarify, with further studies, the mechanism(s) underlying physiological and pathological aspects of male reproduction. This Special Issue confirms, once again, that male reproduction still represents an interesting research area, due to its fundamental contribution to the biological sense of life, which is the continuity of species.
